# 

**Published:** 1852-04

**Authors:** 


Vol. V. APRIL, 1852. No. 3.

### THE DENTAL REGISTER OF THE WEST.

JAMES TAYLOR M. D., D. D. S., EDITOR.

PUBLISHED QUARTERLY;

BY ORDER OF THE MISSISSIPPI VALLEY ASSOCIATION OF DENTAL SURGEONS.

CINCINNATI:
PRINTED BY JOHN D. THORPE,
NO. 74, WEST FOURTH STREET.
1852.

TERMS---$2,00 PER ANNUM, PAYABLE IN ADVANCE.

